# Clinicopathological Correlations in Intracranial Meningiomas

**DOI:** 10.7759/cureus.72723

**Published:** 2024-10-30

**Authors:** Saif A Badran, Alaa A Abdulrazaq, Dahlia R Mohammed, Ahmed A Al-juboori

**Affiliations:** 1 Department of Surgery, Ibn Sina University of Medical and Pharmaceutical Sciences, Baghdad, IRQ; 2 Department of Basic Sciences, Ibn Sina University of Medical and Pharmaceutical Sciences, Baghdad, IRQ; 3 Department of Pathology, Histopathologist, Ministry of Health, Medical City, Teaching Laboratory Complex, Baghdad, IRQ; 4 Department of Neurosurgery, Dr. Sa’ad AL-Witri Hospital for Neurosciences, Baghdad, IRQ

**Keywords:** clinical features, grading, histopathological types, intracranial meningioma, symptomatology

## Abstract

Background: Meningiomas are one of the most common primary intracranial neoplasms that may present with a wide variety of clinical symptoms, depending on multiple factors such as tumor size, location, and grade. Knowledge of the grade of meningioma correlated with their related symptoms is of great value in developing diagnostic and therapeutic approaches efficiently. Although various knowledge about meningiomas exists, there still seems to be a lacuna as far as explicit relations between tumoral characteristics and the severity or evolution of symptoms are concerned. The goal of this prospective study is to describe in detail such associations.

Objective: The goal of this paper is to try to explain the relationship between meningioma grades (I, II, and III) and the spectrum of their clinical manifestations, focusing on the symptom severity and progression dynamics, as well as the anatomical tumor distribution in a series of surgically treated cases.

Materials and methods: In the present prospective study, 117 cases with established diagnoses of intracranial meningioma underwent surgical treatment from January 2021 to January 2024. Detailed analyses of presenting symptoms were done for headaches, seizures, visual disturbances, and motor deficits. Further details in tumor location, such as laterality and regional specificity, and the type of surgical approach adopted were considered in the clinical outcomes. Tumors were also categorized by size (small, large, and huge) to clarify the relationship between tumor size, histologic grade, and clinical symptoms. A correlation between histopathological features and clinical severity based on the grade of tumor was done using immunohistochemistry.

Results: The symptoms presented were, among the three grades, headache in Grade I patients, 54.9%, followed by Grade II, 52.4%, and Grade III accounted for 80.0%. Seizures appeared among 23.8% of the patients in Grade II, whereas among Grade I patients, 15.4% had visual blurring. The size and location of the tumor also differed: the inverted U craniotomy was performed in 32.5% of cases with Grade I and III tumors, whereas the pterional approach was applied more in Grade II and III tumors at 35.9%. In addition, Grade II and III tumors tend to shift to the right side; no obvious asymmetry is seen in Grade I.

Conclusion: The presentation and clinical course of meningiomas seem to be greatly affected and formulated by variables other than meningioma grade, such as tumor location, tumor size, venous blockage (venous sinus/cortical vein), and concomitant perilesional edema. The research emphasizes the necessity for a thorough knowledge of meningioma features for appropriate diagnosis and therapy by highlighting the intricate interaction of various elements in determining symptomatology.

## Introduction

The most common primary intracranial and primary intradural extramedullary spinal tumors in adults are meningiomas. They are graded according to their histopathological characteristics: cellular atypia, mitotic activity, and brain invasion. This grading system is very important in the identification of biological behavior, prognoses, and therapeutic approaches, such as recurrence and the need for adjuvant treatment [1]. Meningioma develops from the arachnoid cells of the leptomeninges and may thus appear wherever these cells are found. Common locations for tumors include the supratentorial compartments (the cerebral convexity, parasagittal area, and sphenoid wing), which greatly affect prognosis and treatment choices (especially surgical resectability) [2].

Recent advances in molecular biology and genetics have shed light on complex processes for the understanding of meningioma, opening up new ways for in-depth study. Indeed, the information provided here increases to some extent the ability to predict clinical outcomes and create tailored therapy for meningioma [3]. Meningiomas are classified, according to WHO criteria, into three groups based on histopathological features. It is this classification, rather than simple grading, that plays a very significant role in guiding diagnostic approaches and treatment options. The classification system does not only predict the biological behavior of the tumor but also answers questions concerning the aggressiveness of treatment. Higher classes, according to WHO, often require more aggressive surgical resections and closer follow-ups due to generally higher recurrence rates [4].

Tumors of the meninges (meningioma) are associated with various clinical and molecular characteristics. Age, gender, radiation exposure, genetics, and race/ethnicity play a role in the presentation of these tumors [5]. Protein mutations, including Merlin/Schwannoma 4, complex genetic alterations that affect atypical and anaplastic meningiomas, have been detected in different areas, such as IB (DAL-1) and protein 4.1R at the molecular level. Deleted chromosomes include 1p, 6q, 10q, 14q, and 18q; there is a duplication of chromosome 17q23. While these breakthroughs are great, the genes driving these changes are still to be identified [6].

The purpose of the study is to prospectively investigate the correlation between meningioma grades and clinical symptoms in terms of symptom intensity and rate of symptom progression. This study investigates the correlations between tumor grade, genetic changes, and symptoms expressed by patients in search of an in-depth look at the multifaceted relationship between meningioma grade, molecular characteristics, and clinical manifestations with critical analyses of existing data.

## Materials and methods

Patient selection

The prospective research included 117 patients with a histological diagnosis of cerebral meningioma from January 2021 to January 2024. Different locations were included, like convexity, parasagittal, falx, and skull base meningiomas, including the temporal tip, olfactory groove, planum sphenoidale, tuberculum sellae, sphenoid wing, foramen magnum, and cerebellopontine angle. Preoperative CT and MRI scans using a variety of sequences, such as T1WI with Gadolinium (axial, coronal, and sagittal views), T2WI, FLAIR, MRA, and MRV, were used for primary diagnosis and surgical planning.

Ibn Sina University of Medical and Pharmaceutical Sciences issued approval ISU.3.2.23.

This analysis selected subjects with only primary meningioma cases. Patients who received previous surgery (redos) or radiation therapy were excluded in order to capture virgin, untreated data.

Surgical procedure and histopathological evaluation

Meningiomas were surgically removed from the brains of all patients using microneurosurgical techniques that involved patient positioning, craniotomy, durotomy, then debasing of the meningioma to control its blood supply and devascularize the meningioma, hence converting it into a less vascular lesion, then debulking of the meningioma (by cutting firstly the dural attached part, then subsequentially removing the tumor going away from its dural attachment) using the open-closed mouth technique with the use of CUSA (Cavitron Ultrasonic Surgical Aspirator), with the exception of small convexity meningiomas that were removed totally in one piece fashion. The histology lab received and sectioned the tumor samples that were taken during the removal procedure. Hematoxylin and eosin stains were used for the histopathological analysis. Immunohistochemical analyses were performed where appropriate, and neuropathologists or histopathologists experienced in neuropathology classified the histopathological findings according to the World Health Organization's 2016 classification of brain tumors.

Histopathological classification

Meningiomas were divided into three WHO grade categories: grade I (which included meningothelial, fibroblastic, transitional, psammomatous, angioid, microcystic, secretory, lymph-plasmacyte-rich, and metaplastic), grade II (which included choroid, clear-cell, and atypical), and grade III (which included papillary, rhabdoid, and anaplastic).

Exclusion criteria

Asymptomatic (incidentally) diagnosed meningiomas were excluded from this study.

Statistical analysis

The data was analyzed by producing summaries in Microsoft Excel 2007. Statistical analyses were conducted using IBM Corp. Released 2017. IBM SPSS Statistics for Windows, Version 25.0. Armonk, NY: IBM Corp., which was chosen due to its reliability and widespread use during the study period. For statistics, the Mann-Whitney U test and the Kruskal-Wallis test were used. The normality check was not required since the data were going to be analyzed using non-parametric tests, and thus, it was omitted. We found the median, minimum, maximum, and quartiles. To compare nominal data sets, we utilized the Freeman-Halton exact test to determine statistical significance. A Bonferroni adjustment was performed to the p-value (α=0.05) to reduce the likelihood of type I errors. A Spearman correlation coefficient was derived to evaluate relationships. The significance level used to determine statistical significance was ≤ 0.05.

## Results

There were 117 patients included in the study's analysis of the sample's demographics. The information showed a median age of 49.0 years, with a minimum age of 16.0 years and a maximum age of 73.0 years. There was a wide variety of ages represented by the participants; the interquartile range, where the 25th and 75th quartiles are located, was determined to be 38.0 years and 60.0 years, respectively.

**Figure 1:**
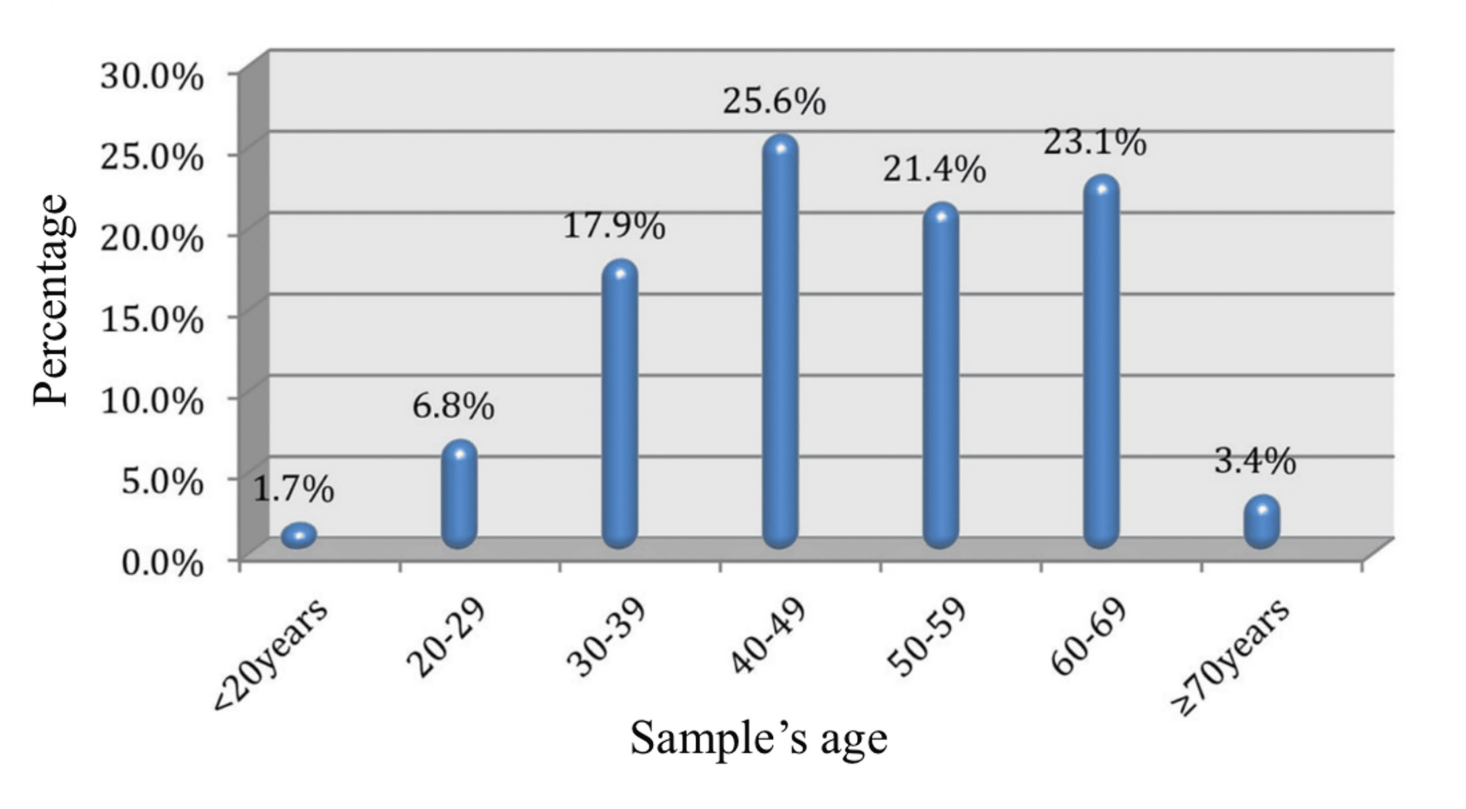
A statistical examination of the sample's age composition is shown in graphical form Data represented as percentages (%).

The results of a statistical examination of the sample's age composition are shown in graphical form in Figure 1. The findings showed that those between the ages of 40 and 49 made up the largest age group (25.6% of the overall sample).

The next largest demographic was made up of those aged 60-69 (23.1%) (27 out of 117), then those aged 50-59 (21.4%) (25 out of 117). Significantly, 6.8% (eight out of 117) of the sample consisted of people aged 20 to 29. Comparatively, the research found far lower rates among individuals aged 20 years, 1.7% of the whole sample (two out of 117), and 70 years, 3.4% of the total sample (four out of 117).

**Figure 2:**
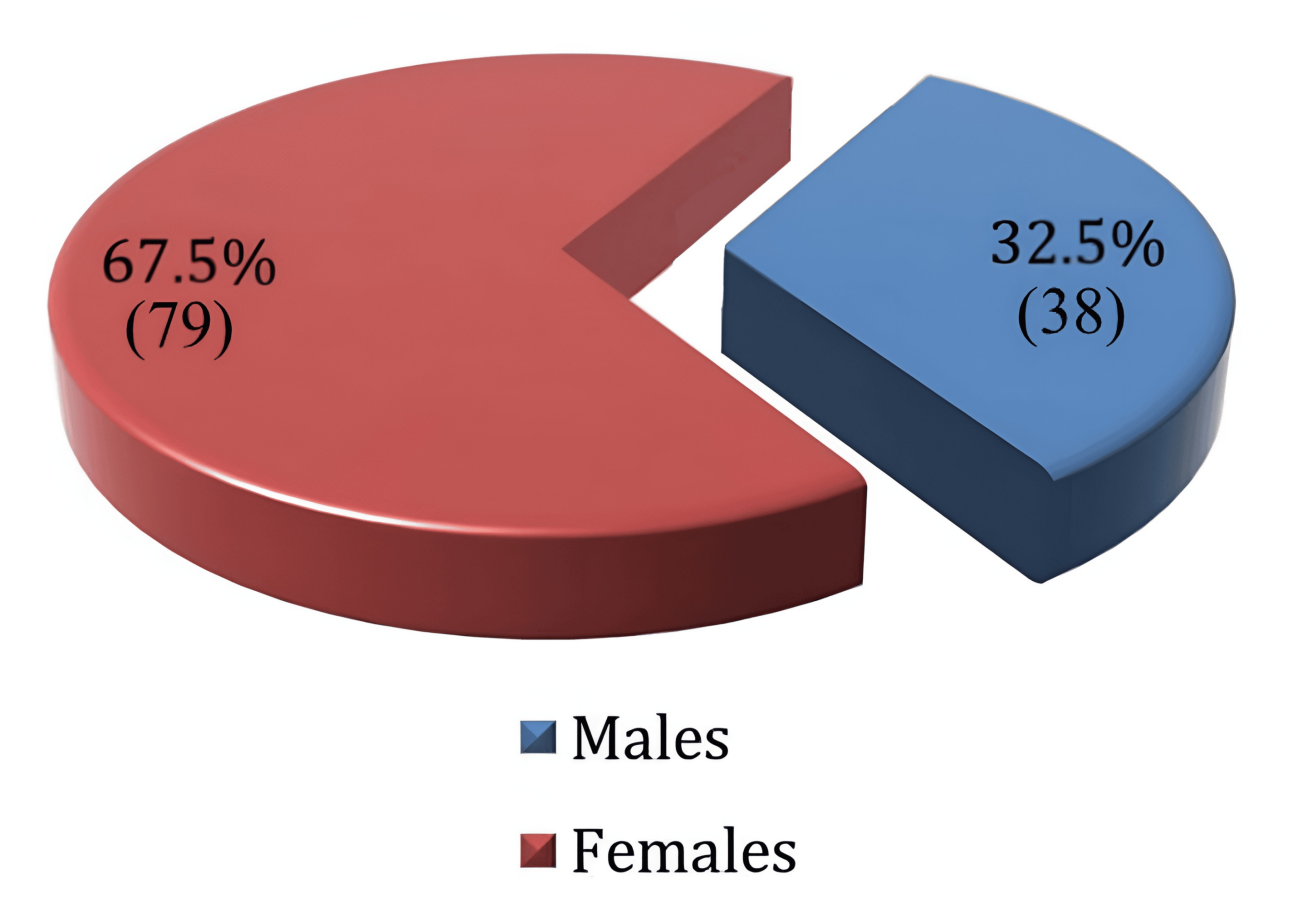
The distribution of the study sample by gender Data represented as N (%).

Figure 2 displays the results of an in-depth review of the research data pertaining to the distribution of the study sample by gender. A total of 117 patients were included in the study, with 38 (32.5%) self-identifying as male, while 79 (67.5%) were female.

The distribution of cases was tabulated in Table 1 within the WHO tumor grades. The highest distribution was found in the 40-49 age group, accounting for 26.3%. Grade III tumors were higher in this age group (40%), and Grade II tumors were more frequent in the 60-69 age group with 28.6%.

**Table 1:** The comparison of age groups among the WHO grades Data is represented as N (%). Statistical significance is determined by (p < 0.05).

Age groups	Grades			Total	p-value
	I (n=91) No. (%)	II (n=21) No. (%)	III (n=5) No. (%)		
<20years	2(2.2)	0(0.0)	0(0.0)	2(1.7)	0.844*
20-29	6(6.6)	1(4.9)	1(20.0)	8(6.8)	
30-39	17(18.9)	4(19.0)	0(0.0)	21(17.9)	
40-49	24(26.3)	4(19.0)	2(40.0)	30(25.6)	
50-59	20(21.9)	4(19.0)	1(20.0)	25(21.4)	
60-69	20(21.9)	6(28.6)	1(20.0)	27(23.2)	
≥70years	2(2.2)	2(9.5)	0(0.0)	4(3.4)	

Table 2 from the research shows some intriguing gender-specific tendencies within each tumor grade based on an analysis of the gender distribution across WHO grades.

**Table 2:** The comparison of gender among the WHO grades Data is represented as N (%). Statistical significance was determined using the Freeman-Halton Exact Test (p < 0.05).

Gender	Grades			Total	p-value
	I (n=91) No. (%)	II (n=21) No. (%)	III (n=5) No. (%)		
Males	31(34.1)	7(33.3)	0(0.0)	38(32.5)	0.325*
Females	60(65.9)	14(66.7)	5(100.0)	79(67.5)	

As a consequence of this trend, gender has been proposed as an important factor in tumor grading. In terms of the distribution of tumor grades between sexes, the p-value of 0.325 indicates that there are no statistically significant differences.

The symptoms associated with each WHO grade are detailed in Table 3, which provides a comprehensive comparison of the varying clinical presentations across all WHO grades. The data shows that headache is the most common first symptom, occurring in 54.9% (50 out of 91) of grade I cases, 52.4% (11 out of 21) of grade II cases, and 80.0% (4 out of 5) of grade III cases.

**Table 3:** The comparison of presentations among WHO grades Data is represented as N (%). Statistical significance was determined using the Freeman-Halton Exact Test (p < 0.05).

Presentations	Grades			Total	p-value
	I (n=91) No. (%)	II (n=21) No. (%)	III (n=5) No. (%)		
Headache	50(54.9)	11(52.4)	4(80.0)	65(55.5)	0.833*
Seizure	18(19.8)	5(23.8)	0(0.0)	23(19.7)	
Blurred vision	14(15.4)	3(14.3)	0(0.0)	17(14.5)	
Motor Weakness	9(9.9)	2(9.5)	1(20.0)	12(10.3)	

Furthermore, 19.8% (18 out of 91) of grade I cases and 23.8% (5 out of 21) of grade II cases were linked to seizures, while none of the grade III cases were. The prevalence of visual impairment was also higher in grade I (15.4%) (14 out of 91) than in grade II (14.3%) (3 out of 21). Significantly, 10.3% (12 out of 117) of the whole patient sample showed signs of weakening, with 9.9% (9 out of 91) showing grade I, 9.5% (2 out of 21) showing grade II, and 20.0% (1 out of 5) showing grade III.

A careful statistical analysis was performed to determine the significance of these findings, and a p-value of 0.833 was calculated. There are no statistically significant variations in clinical presentation across the several WHO grades, as shown by this value.

**Figure 3:**
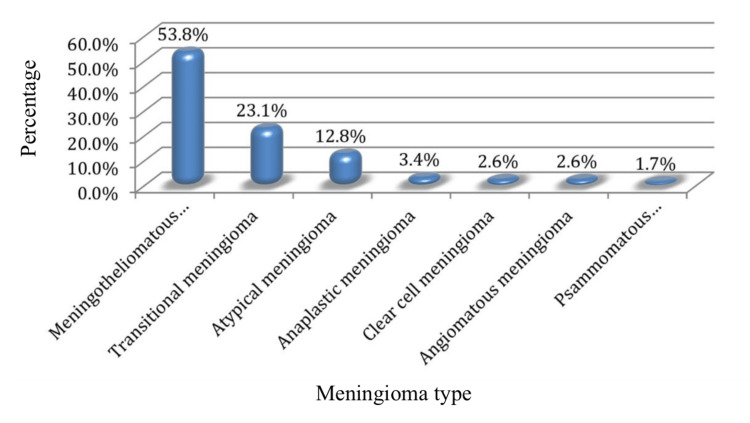
Meningioma types within the study Data are represented as percentages (%). Statistical significance was determined using the Freeman-Halton Exact Test (p < 0.05).

Figure 3 presents a thorough study of meningioma types within the examined cohort, elucidating the many histological classifications seen. The meningotheliomatous subtype of meningioma has been shown to account for 53.8% (63 out of 117) of all cases. Next came transitional meningioma, accounting for 23.1% (27 out of 117) of diagnoses, followed by atypical meningioma at 12.8% (15 out of 117). In particular, diagnoses of meningioma were made in 2.6% (3 out of 117) of cases for anaplastic meningioma, 2.6% (3 out of 117) for clear cell meningioma, and 1.7% (2 out of 117) for psammomatous meningioma.

**Figure 4:**
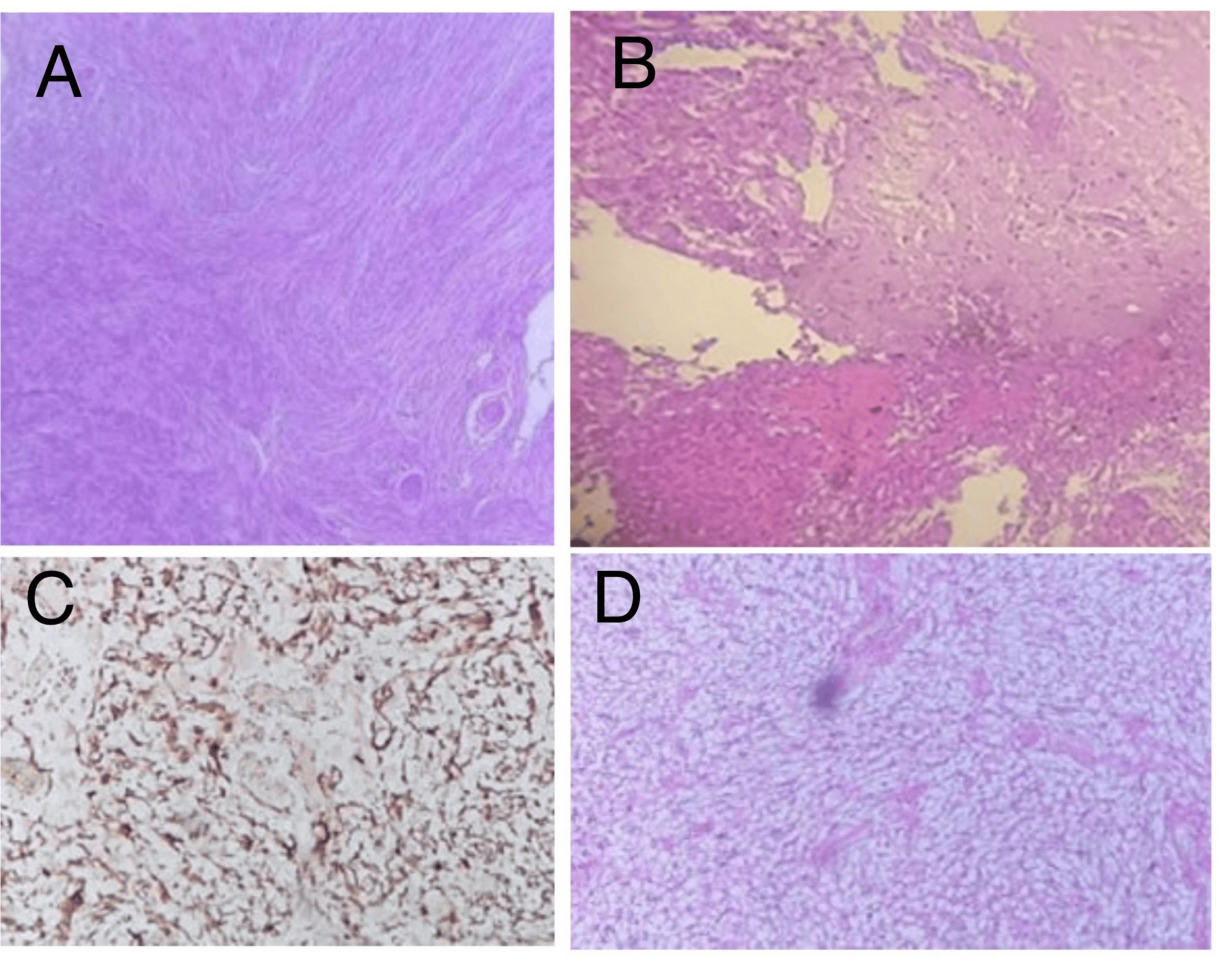
Histopathological findings showing: A: Transitional meningioma WHO grade I tumor H&E stain (×10). B: WHO grade II atypical meningioma glial tissue infiltrated by neoplasm. H&E stained slide (×10). C: Clear cell meningioma IHC positive for vimentin (same previous case). D: Clear cell meningioma, WHO grade II, H&E stained slide (×10).

**Figure 5:**
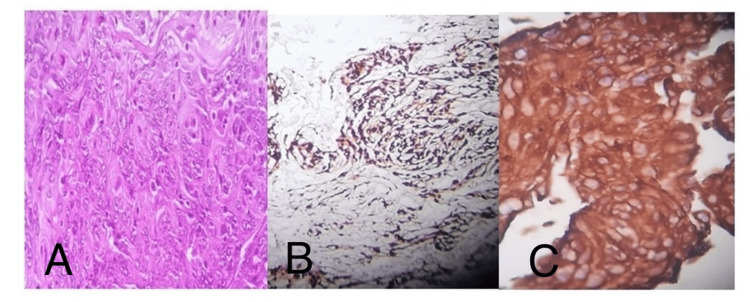
Histopathological findings showing: A: WHO grade II atypical meningioma with increased cellularity, prominent nucleoli, and foci of sheet-like pattern, H&E-stained slide (X40). B: Atypical meningioma with positive nuclear staining for PR. C: Atypical meningioma positive for vimentin (positive membrane staining).

**Figure 6:**
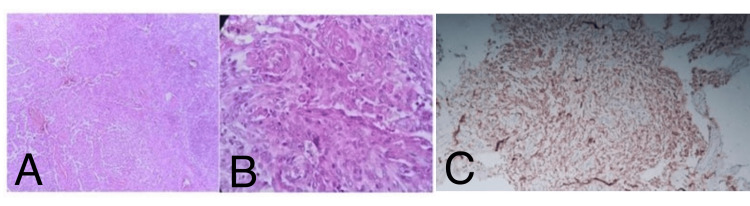
Histopathological findings showing A, B: Meningotheliomatous meningioma WHO grade I tumor, H&E stain (x10 & x40), C-Clear cell meningioma, WHO grade II, IHC positive for EMA

This fine-grained classification of meningioma types shows the histological variety of the patient population. Meningioma subtype prevalence information is crucial for proper diagnosis and subsequent therapy planning (Figures 4-6).

Table 4 demonstrates the comparison of surgical approaches used among WHO grades. It shows that there is no significant statistical difference among the grades (p=0.665). However, the pterional approach appears as the most frequent approach used among the sample 42 (34.9%), followed by inverted U craniotomy 38 (32.5%), lateral supra-orbital approach 27 (23.5%), and retrosigmoid approach 7 (5.5%). The sub-occipital approach, bi-frontal trans-basal approach, and far lateral trans-condylar approach are performed in only one patient for each. For the WHO grades, inverted U craniotomy is the most frequent surgical approach for grade I and grade III, while the pterional approach is most frequently used for grade II. 

**Table 4:** The comparison of surgical approaches among WHO grades Data is represented as N (%). Statistical significance was determined using the Freeman-Halton Exact Test (p < 0.05).

Surgical approach	Grades			Total	p-value
	I (n=91) No. (%)	II (n=21) No. (%)	III (n=5) No. (%)		
pterional	29(31.8)	12(57.1)	1(20.0)	42(35.9)	0.665*
inverted U craniotomy in unsuspected locations	31(34.1)	5(23.8)	2(40.0)	38(32.5)	
lateral SOA	23(25.3)	3(14.3)	1(20.0)	27(23.2)	
Retro-sigmoid approach	5(5.5)	1(4.8)	1(20.0)	7(5.5)	
Sub-occipital approach	1(1.1)	0(0.0)	0(0.0)	1(0.8)	
Bi-frontal trans-basal approach	1(1.1)	0(0.0)	0(0.0)	1(0.8)	
far lateral trans-condylar approach	1(1.1)	0(0.0)	0(0.0)	1(0.8)	

Meningiomas' geographical distribution may be better understood with the help of Table 5, which gives a detailed analysis of tumor sites across various WHO grades. The research shows that there are very small changes in likely locations among grades, with the exception of a few places where large discrepancies were seen.

**Table 5:** Comparison of the location among the WHO grades Data is represented as N (%). Statistical significance determined using the Freeman-Halton Exact Test (p < 0.05)

Geographic distribution (site)	Grades			Total	p-value
	I (n=91) No. (%)	II (n=21) No. (%)	III (n=5) No. (%)		
Medial sphenoid wing (Clinoidal)	9(9.9)	2(9.5)	1(20.0)	12(10.3)	0.762
Middle sphenoid wing	6(6.6)	2(9.5)	0(0.0)	8(6.8)	0.735
Lateral sphenoid wing	1(1.0)	0(0.0)	0(0.0)	1(0.09)	0.865
Cerebello-pontine angle	6(6.6)	0(0.0)	0(0.0)	6(5.1)	0.405
Cavernous sinus	2(2.2)	0(0.0)	0(0.0)	2(1.7)	0.747
Petro-clival and parasellar	1(1.0)	0(0.0)	0(0.0)	1(0.09)	0.865
Olfactory groove	11(12.1)	1(4.8)	0(0.0)	12(10.3)	0.451
Tuberculum sellae	10(11.0))	1(4.8)	1(20.0)	12(10.3)	0.533
Planum sphenoidale	0(0.0)	1(4.8)	0(0.0)	1(0.09)	0.099
Frontal skull base	2(2.2)	0(0.0)	0(0.0)	2(1.7)	0.747
Foramen magnum	1(1.0)	0(0.0)	0(0.0)	1(0.09)	0.865
Orbital roof	2(2.2)	0(0.0)	0(0.0)	2(1.7)	0.747
Lateral ventricular	3(3.3)	0(0.0)	0(0.0)	3(2.6)	0.644
Frontal convexity	2(2.2)	0(0.0)	0(0.0)	2(1.7)	0.747
Parietal convexity	9(9.9)	6(28.6)	0(0.0)	15(12.8)	0.052
Fronto-parietal convexity	1(1.0) ^A^	4(19.0) ^B^	0(0.0) ^AB^	5(4.3)	0.001
Fronto-temporal convexity	3(3.3)	0(0.0)	0(0.0)	3(2.6)	0.644
Temporal convexity	1(1.0)	2(9.5)	0(0.0)	3(2.6)	0.082
Occipital convexity	1(1.0)	0(0.0)	0(0.0)	1(0.09)	0.865
Anterior falcine	4(4.4)	2(9.5)	0(0.0)	6(5.1)	0.547
Posterior falcine	3(3.3)	0(0.0)	0(0.0)	3(2.6)	0.644
Frontal parasagittal	4(4.4)	0(0.0)	1(20.0)	5(4.3)	0.137
Fronto-parietal parasagittal	3(3.3)	0(0.0)	0(0.0)	3(2.6)	0.644
Occipital parasagittal	1(1.0)	0(0.0)	0(0.0)	1(0.09)	0.865
Temporal base	1(1.0)	0(0.0)	0(0.0)	1(0.09)	0.865
Temporal tip	1(1.0) ^A^	0(0.0) ^A^	2(40.0) ^B^	3(2.6)	0.000
Falco-tentorial	2(2.2)	0(0.0)	0(0.0)	2(1.7)	0.747
Tentorial	1(1.0)	0(0.0)	0(0.0)	1(0.09)	0.865

The statistically significant variance was seen in instances of frontoparietal convexity meningioma (p=0.001). After accounting for Bonferroni's correction, the difference between grades I and II is not statistically significant; 19.0% (4 out of 21) of grade II patients displayed this location. Curiously, this area was only detected in grade II patients, underscoring the peculiarity of that grade location. Similarly, a statistically significant difference was seen with temporal tip meningioma. Bonferroni's corrected analysis revealed that grade III was much more common than grades I and II, with 40.0% (2 out of 5) of instances compared to 1.0% (1 out of 91) and 0% (0 out of 21), respectively.

Table 6 presents an in-depth examination of the correlation between the dominant lesion side and WHO grade for all meningioma subtypes. The data shows that grade I meningiomas are distributed evenly across the left and right sides of the head. Grade II meningiomas showed a marginal preference for the right side (55.6% of cases) (10 out of 18), whereas grade III meningiomas showed a much more pronounced right-side dominance (75.0% of cases) (3 out of 4).

**Table 6:** Comparison of the dominant side among the WHO grades Data is represented as N (%). Statistical significance was determined using the Freeman-Halton Exact Test (p < 0.05).

The side of meningioma	Grades			Total	p-value*
	I (n=68) No. (%)	II (n=18) No. (%)	III (n=4) No. (%)		
Left dominant sided	34(50.0)	8(44.4)	1(25.0)	43(47.8)	0.671
Right-sided	34(50.0)	10(55.6)	3(75.0)	47(52.2)	

A p-value of 0.671 indicates that there is no statistically significant variation in the side distribution of lesions between grades. However, these results do give useful clinical insights into the laterality patterns of meningiomas across all WHO categories. Therefore, they should not be discounted just because they lack statistical significance. Although statistical significance may not always be apparent, knowing which side these lesions tend to develop on is essential for careful preoperative planning and possible prognostic consequences.

Table 7 provides a detailed examination of the associations between WHO grades and various important factors in the research, such as age, gender, location of meningiomas, clinical presentations, and surgical methods. According to the data analysis, no significant relationships can be seen between WHO grades and any of these factors. In this study's cohort of patients with meningiomas, no statistically significant associations were found between demographic variables and WHO grade.

**Table 7:** The correlation of WHO grades with study variables Data are represented as correlation coefficients (r). Statistical significance was determined using the Spearman correlation coefficient (p < 0.05). a. Not assuming the null hypothesis. b. Using the asymptotic standard error, assuming the null hypothesis. c. Based on normal approximation.

Spearman Correlation	r	Asymp. Std. Error^a^	Approx. T^b^	p-value
Age	0.056	0.096	0.596	0.552^c^
Gender	0.076	0.086	0.819	0.414^c^
Sites	0.103	0.096	1.112	0.269^c^
Presentations	-0.025	0.092	-0.267	0.790^c^
Operation approach	0.118	0.094	1.276	0.205^c^

These findings indicate that the WHO classification for grading meningiomas seems unaffected by the factors evaluated here. Considering the complexity and multiple nature of meningioma formation and progression, the absence of a substantial association is to be expected. In spite of their undeniable impact in the clinical setting, our study did not find a statistically significant link between the characteristics above and the WHO-defined histopathological grade.

## Discussion

In this research, we examined a group of 117 individuals with meningioma, concentrating on several clinicopathological features and their consequences. Our results are consistent with the literature already available; for example, the median age of 49 years in our cohort is similar to that found in the study by Alaa Ghani Hussein et al. [7]. However, it is different from the report by Ostrom Q.T. et al. This variation highlights the complex interplay of several elements in meningioma formation, including, but not limited to, mutagenic exposure and hormonal regulation [8].

Our results showed a strong preference for females (67.5%) (79 out of 117), which is in line with previous studies [9-11]. Hormonal factors may play a role in meningioma development since these tumors express hormone receptors such as somatostatin receptor 2 (SSTR2), progesterone, estrogen, and androgen. Intriguingly, the majority of our cases were WHO grade I (77.8%) (91 out of 117), similar to the findings in Germany [12] and the global analysis by Louis D.N. et al. [13].

The results confirmed Alaa Ghani Hussein's findings that meningotheliomatous meningioma was the most common histological subtype (53.8%) [7]. The absence of substantial associations between age, gender, and tumor grade in our study is consistent with the findings of Sarah Ali Abed [9]. However, higher-grade tumors were shown to rise with patient age, which may be attributable to younger patients' heightened awareness leading to early medical intervention.

There was wide variation in the clinical presentation, with headaches being the most prevalent complaint (55.5%) (65 out of 117), which is in line with earlier research [7]. When compared to the findings of Hussein et al., the prevalence of seizures (19.7%) was much lower [7]. The relevance of seizures in identifying atypical meningiomas was highlighted by the fact that they were most often linked with WHO grade II tumors (23.8%) (5 out of 21). Since weakness is often associated with tumor size and location, its linkage with meningioma grades needs to be evaluated more thoroughly.

The pterional approach (35.9%) (42 out of 117) that was originally described and popularized by Yasargil in the 1970s to address the treatment of anterior communicating artery aneurysms [14] and can be used for different kinds of meningiomas, namely the anterior skull base meningioma, was the most prevalent of the tumor-specific surgical procedures. Additionally, 32.5% of all craniotomies were in the form of an inverted U, often performed on patients with convexity, parasagittal, or falcine meningiomas. These methods highlight the fact that meningioma procedures are highly individualized.

Parietal convexity was found to be the most common location (12.8% of the time, 15 out of 117). In contrast to the findings of Gervásio Teles et al., who found that some locations were linked to high mortality [15], we found no statistically significant differences across sites with respect to WHO grades, contrary to the finding of Pereira B.J.A. et al., who found that the skull base meningioma is more likely to be grade I [16]. Separating lesions into those on the dominant left and those on the non-dominant right revealed interesting patterns, but more thorough research is needed to reach firm conclusions.

In conclusion, despite the fact that there are no significant associations between certain clinicopathological criteria and tumor grades, the importance of early discovery, thorough surgical planning, and complete follow-up cannot be overstated in the treatment of meningiomas.

Over the last decade, meningioma behavior has been increasingly understood in the context of an evolving complex molecular underpinning for these tumors. In the past, WHO grade I meningiomas were considered to be benign lesions, but there have been a number of recurrences and malignant transformations, which point to a rather molecular understanding or even radiating the residual lesions postoperatively. Surgical resection remains a cornerstone in the management of meningiomas; indeed, the extent of resection has been playing a critical role in predicting outcomes. However, even after gross total resection, recurrence rates remain high, especially for higher grades of meningioma. Molecular markers such as NF2 mutations, copy number variations, and epigenetic modifications have further supported the fact that heterogeneity exists in meningioma behavior and that not every case should be treated with the same approach. These findings further emphasize the integration of molecular data in surgical decision-making for meningioma patients to provide appropriate treatment strategies and to optimize long-term outcomes [17].

Meningiomas are often managed with a staged surgical approach, especially for complex cases in which either the tumor is large or the tumor is situated near critical neurovascular structures. This technique allows a much safer and more controlled resection process while minimizing risks of brain retraction and excessive blood loss. One of the most salient benefits derived from staging a resection is that it first affords the opportunity to devascularize the tumor, reducing its size and vascularity and making subsequent resections less challenging by giving extra space to manipulate the tumor while decreasing manipulation of the critical neural structures. Gendreau et al. [18] state that factors generally involved in indications for staging include tumor size, tumor location, and proximity to essential neurovascular structures. Generally, the approach is well-tolerated and with few complications; however, additional studies are necessary to develop a systematic approach to its application. This paper emphasizes the need for standardized guidelines so as to improve clinical decision-making in the surgical management of difficult-to-treat meningiomas.

The sample size is 117 cases; hence, generalization to bigger populations may be limited. A single-center design brings location-based biases, and therefore universal application may not be feasible. The lack of longitudinal data further limits charting long-term symptom progression. Last but not least, there was little discussion of the variability in technique applied in the surgeries, which may subsequently result in differences in results.

One of the inherent limitations in this study is the stratification of patients according to their most prominent symptoms, which could miss the complexity brought about by multiple presentations of symptoms. This was done to enable statistical analysis; however, further studies may consider a different approach that can accommodate more than one symptom manifestation for each patient.

## Conclusions

This present study emphasizes the complexity of meningiomas, pointing out that the timing of early detection and treatment should be individualized according to tumor location, size, and histopathological features. Factors that will also guide the clinical approach to determine prognosis. There was no significant association between demographic variables and tumor grade, but symptomatology and surgical outcomes did show trends in this study, indicating a need for further individualization of patient management. Exploration of the interrelationship between molecular changes and tumor behavior is an indication for further studies that provide additional insights into treatment strategies and long-term outcomes.
